# The Guardian of Health; a Monthly Journal of Domestic Hygiene

**Published:** 1842-02

**Authors:** 


Art. IV.—*The Guardian of Health; a monthly Journal of Domestic Hygiene.*

This is the title of a new periodical commenced at Baltimore in September last, and of which the first, second, and third numbers have been published. It is under the editorial charge of Thomas E. Bond, Jr. M. D., and Chapin A. Harris, M. D. The design of the publication is made known in a very well written prospectus, from which we make the following quotations.

"The Guardian of Health is not intended, and will not be calculated, to supersede the physician. Its object will be to prevent disease and suffering by such precautionary information as may appear necessary. It will aid the mother in the performance of her proper duties as guardian of her children's health—it will aim to make her a skilful nurse, and a judicious, firm, and tender assistant to the physician in the management of disease."

"The care of the teeth and the teething infant is a matter of very great importance. Parents are not aware how often death in dentition, or the ill health of subsequent life, is the result of improper management during the first months of infancy, nor do they suspect the great instrumentality which imperfect development, or serious disorders of the organs of the mouth, exert in the production of depraved and debilitated constitutions. Mothers to whom the care of these things almost exclusively belong are necessarily ignorant about them. But little effort has been made to afford them information, and even when alive to the subject, they do not know where to obtain the necessary knowledge.

It is hoped that the journal now proposed will supply this important aid. A portion of it will be regularly devoted to information upon the hygiene of dentition, and the care of the teeth and their appendages."

One thing is adverted to in this prospectus that cannot have escaped the notice of our readers: we allude to the great number of deaths among children, as shown by our bills of mortality. The fact is beyond all question. We happen to have before us, at this moment, the "Interments in the City and Liberties of Philadelphia, from the 4th to the 18th of December, 1841." The whole number is 188, of which 90, or almost one-half, are children. This is indeed painful, and in the language of the editors, "no experienced physician can doubt that much of this premature mortality, with its fearful train of domestic misery, is unnecessary."

Undoubtedly, much of it is owing to a want of energy in the treatment of the diseases incident to childhood. The practice is too often an *expectant* one. Physicians are frequently heard to remark that they "do not like to give medicine to children." Of course not; but it would be much more harmless, and quite as true, to say, that "children do not like to take medicine." This, besides, would have the advantage of not affording to parents and nurses any pretext or countenance for delay in seeking advice, and for tampering with disease until it is beyond remedy. Rush's great maxim to "oppose the beginnings of disease," if valuable at all, is peculiarly so in the management of children, and it should ever be kept in mind.

The objects of the "Guardian" are certainly praiseworthy, and we accordingly give the enterprise our best wishes.

